# Clinical Characteristics and Outcomes of Older Patients Admitted to the Cardiac Intensive Care Unit

**DOI:** 10.1016/j.jacadv.2026.102830

**Published:** 2026-06-17

**Authors:** Constantine Tarabanis, Jianping Guo, Gregory W. Barsness, Maryam Farahmandsadr, Christopher B. Fordyce, Michael Goldfarb, Jason N. Katz, Michael C. Kontos, P. Elliott Miller, L. Kristin Newby, Sean van Diepen, David A. Morrow, Carlos L. Alviar

**Affiliations:** a Cardiology Division, Heart and Vascular Institute, Mass General Brigham, Boston, Massachusetts, USA; b Levine Cardiac Intensive Care Unit, Cardiovascular Division, Department of Medicine, Brigham and Women’s Hospital, Harvard Medical School, Boston, Massachusetts, USA; c Division of Critical Care Cardiology, Department of Cardiovascular Medicine, Mayo Clinic, Rochester, Minnesota, USA; d Division of Cardiology, University of Florida College of Medicine, Jacksonville, Florida, USA; e Division of Cardiology, Vancouver General Hospital, and the Center for Cardiovascular Innovation, University of British Columbia, Vancouver, British Columbia, Canada; f Division of Cardiology, Jewish General Hospital, McGill University, Montreal, Quebec, Canada; g Division of Cardiology, New York University Grossman School of Medicine & Bellevue Hospital, New York, New York, USA; h Division of Cardiology, Virginia Commonwealth University, Richmond, Virginia, USA; i Section of Cardiovascular Medicine, Yale School of Medicine, New Haven, Connecticut, USA; j Division of Cardiology, Department of Medicine, Duke Clinical Research Institute, Duke University Medical Center, Durham, North Carolina, USA; k Department of Critical Care Medicine and Division of Cardiology, Department of Medicine, University of Alberta, Edmonton, Alberta, Canada

**Keywords:** cardiac arrest, cardiac intensive care unit, cardiogenic shock, critical care cardiology, geriatrics, in-hospital mortality

## Abstract

**Background:**

Contemporary data characterizing older adults admitted to cardiac intensive care units (CICUs) across diverse indications are limited.

**Objectives:**

The objective of the study was to describe the clinical characteristics, critical care therapies, and in-hospital outcomes of older patients admitted to the CICU compared with younger adults.

**Methods:**

The Critical Care Cardiology Trials Network is a multicenter, international registry of CICUs. Between 2017 and 2024, participating centers contributed annual ≥2-month snapshots of consecutive medical CICU admissions. Admissions were categorized into 4 age groups: <65, 65–<75, 75–<85, and ≥85 years. Outcomes included CICU and in-hospital mortality and length of stay. Multivariable models adjusted for sex, illness severity (SOFA score), lactate, and kidney function.

**Results:**

Among 35,265 admissions from 50 sites, 44%, 27%, 21%, and 9% were aged <65, 65–<75, 75–<85, and ≥85 years, respectively. Acute coronary syndrome was the most common admission diagnosis among all age groups. Patients aged ≥85 years had the lowest use of mechanical circulatory support (5.5%), which consisted exclusively of intra-aortic balloon pumps. Relative to patients <65 years, adjusted ORs of in-hospital mortality were 1.53 (1.40-1.67) for 65–<75 years, 1.83 (1.67-2.01) for 75–<85 years, and 1.95 (1.72-2.22) for ≥85 years. Among cardiac arrest patients the increase in mortality with age was steeper, reaching 3.09 (2.24-4.26) for patients ≥85 years.

**Conclusions:**

Patients ≥85 years in contemporary CICUs experience survival comparable to those aged 75–<85 years, except in the setting of cardiac arrest. These findings support consideration of factors beyond chronological age in CICU triage and treatment decisions.

The aging of the population along with the increased prevalence of cardiovascular (CV) disease (CVD), has translated into a greater number of older patients requiring hospital admissions to treat CV conditions.^1^ It is estimated that approximately 85% of patients above the age of 80 years have CVD, with CVD-related hospitalization rates increasing among older men and women.^2^^,^^3^ Moreover, geriatric syndromes such as frailty, cognitive decline, impaired mobility, multimorbidity, and polypharmacy contribute to the higher risk profile and mortality in older (>65 years) patients with CVD.^4^^,^^5^ Such factors may increase severity of illness and burden of comorbidity accompanying CVD.^6^ This shifting epidemiology is likely to have an ongoing impact on cardiac intensive care units (CICUs).

Despite this ongoing demographic and epidemiological shift, published data on the clinical characteristics and outcomes of older patients admitted to the CICU remain limited. The few prior studies of this population have mostly focused on specific age subsets, including more extreme age ranges (eg, nonagenarians),^7^^,^^8^ and lacked comparisons with patients aged 65 to 85 years old, and their subgroups.^9^, ^10^, ^11^ In addition, such studies have largely focused on specific disease processes or interventions, such as acute coronary syndrome (ACS)^12^ or percutaneous coronary intervention (PCI),^13^, ^14^, ^15^ with smaller studies evaluating other high acuity conditions such as cardiogenic shock (CS).^16^ Thus, there is a knowledge gap regarding the clinical characteristics and patterns of practice among critically ill cardiac patients who are 65 years of age or older (henceforth referred to as “older” patients). We aimed to characterize the clinical features, critical care therapies, and in-hospital survival among older patients admitted to the CICU compared with younger (<65 years) patients. With this analysis, we sought to describe the association between age and CICU admission, prognosis, and treatment decisions in older patients.

## Methods

### Study population

The Critical Care Cardiology Trials Network is an investigator-initiated, collaborative network of predominantly American Heart Association level I CICUs coordinated by the TIMI Study Group (Boston, MA).^17^ The Critical Care Cardiology Trials Network Registry design and data collection methods have been described.^18^ Participating centers contributed clinical data on all consecutive medical admissions to the CICU during annual minimum 2-month collection periods. Medical CICU admissions were included in the analysis cohort, omitting general medical ICU overflow and routine postcardiac surgical admissions. To minimize the risk of patient reidentification, age in the case report form was capped at 90 years, in alignment with common deidentification standards. Each center’s Institutional Review Board approved the study protocol and, with only deidentified data collected in the registry, waived informed consent.

### Data collection

Clinical data were collected through a comprehensive standardized clinical review of each patient.^18^ Clinical and demographic characteristics were obtained from electronic medical records. Measures of illness severity included the sequential organ failure assessment (SOFA) score and the worst laboratory values within 24 hours of CICU admission (using previously reported definitions).^19^ Outcomes of interest included in-hospital and CICU mortality and length of stay (LOS).

### Statistical analysis

CICU admissions were categorized into 4 age groups: <65, ≥65 to <75, ≥75 to <85, and ≥ 85 years based on patient age at the time of CICU admission.^9^, ^10^, ^11^ Values are presented as medians (25th, 75th percentiles) for continuous variables and counts with percentages (%) for categorical variables, unless otherwise specified. The Kruskal-Wallis test was used for comparisons of continuous variables. Chi-square tests were used for comparisons of categorical variables. Trends across campaigns were evaluated using the Cochran-Armitage trend test. Multivariable linear and logistic regression were used to adjust for potential confounders including sex, SOFA score, lactate concentration, and estimated glomerular filtration rate (eGFR).

To further assess for potential confounding, an expanded multivariable model was performed as a sensitivity analysis, incorporating additional chronic comorbidities including diabetes mellitus, significant pulmonary disease, and significant liver disease. To account for potential informative censoring due to differences in LOS, a sensitivity analysis was performed using multivariable Cox proportional hazards regression to evaluate the association between age and the timing of in-hospital mortality. Primary analyses were conducted using observed data without imputation of missing values. Missingness was 0% for age, SOFA score, and comorbidities, and 0% for eGFR. Lactate was missing in 35% of cases and was classified as <2 mmol/L for the primary analysis. Sensitivity analyses were performed using complete case analysis and multiple imputation by fully conditional specification (20 data sets) including age, sex, race, and hospital mortality as predictors. Analyses were performed using SAS software (version 9.4; SAS Institute Inc.). A 2-sided *P* ≤ 0.05 was considered statistically significant.

## Results

### Baseline characteristics

A total of 35,265 medical CICU admissions from 2017 to 2024 across 50 sites were included in this analysis, with 15,472 (44%) in the <65, 9,431 (27%) in the 65 to <75, 7,293 (21%) in the 75 to <85, and 3,069 (9%) in the ≥85 age groups. Table 1 describes the baseline demographic and clinical characteristics by these age groupings. All demographic characteristics differed among the 4 age groups (Table 1). The ≥85-year age group had the highest proportion of White (79.5%) and female (49.8%) patients, as well as the lowest body mass index (Table 1). The frequency of most cardiac and noncardiac comorbid conditions also differed by age group. Patients aged ≥85 years had the highest proportion of cerebrovascular disease (12.9%), atrial fibrillation (38.9%), and severe valvular disease (27.2%) (Table 1). Over time, the cohort became progressively older, with patients ≥65 years comprising 52.7% of admissions in 2017 increasing to 57.3% in 2023 (Supplemental Table 1) (*P* for trend *< 0.0001*).

**Table 1:** Baseline Demographic and Clinical Characteristics of the Analysis Population (N = 35,265) Stratified by Age Group

	Age <65 y (n = 15,472)	Age ≤65 y to <75 y (n = 9,431)	Age ≤75 y to <85 y (n = 7,293)	Age ≥85 y (n = 3,069)
Demographics				
Age, median (25th, 75th percentiles), y	55.0 (46.0-60.0)	69.0 (67.0-72.0)	79.0 (77.0-82.0)	88.0 (86.0-89.0)
Female	4,997 (32.3%)	3,371 (35.7%)	2,960 (40.6%)	1,527 (49.8%)
White race	7,496 (60.0%)	5,461 (70.8%)	4,439 (75.4%)	1,964 (79.5%)
BMI, median (IQR), kg/m^2^	28.3 (24.4-33.5)	27.7 (24.2-32.3)	26.9 (23.5-30.8)	25.1 (22.2-28.4)
Cardiovascular history				
Current smoker	3,812 (27.6%)	1,380 (16.5%)	517 (8.1%)	80 (3.0%)
Diabetes mellitus	4,513 (29.2%)	3,843 (40.7%)	2,862 (39.2%)	899 (29.3%)
Hypertension	7,856 (50.8%)	6,430 (68.2%)	5,481 (75.2%)	2,370 (77.2%)
Coronary artery disease	3,758 (24.3%)	3,880 (41.1%)	3,336 (45.7%)	1,343 (43.8%)
Cerebrovascular disease	971 (6.3%)	899 (9.5%)	801 (11.0%)	396 (12.9%)
Peripheral artery disease	874 (5.6%)	1,090 (11.6%)	968 (13.3%)	350 (11.4%)
Prior heart failure	5,393 (34.9%)	3,591 (38.1%)	2,838 (38.9%)	1,136 (37.0%)
Historical LVEF				
≥50%	940 (18.1%)	883 (25.4%)	977 (36.0%)	510 (46.8%)
<50%	4,262 (81.9%)	2,592 (74.6%)	1,736 (64.0%)	579 (53.2%)
Atrial fibrillation	2,710 (17.5%)	2,631 (27.9%)	2,500 (34.3%)	1,193 (38.9%)
Ventricular arrhythmia	1,127 (7.3%)	660 (7.0%)	385 (5.3%)	91 (3.0%)
Severe valvular disease	1,422 (9.2%)	1,347 (14.3%)	1,483 (20.3%)	834 (27.2%)
Noncardiovascular comorbidities				
Chronic kidney disease	2,864 (18.5%)	2,427 (25.7%)	2,236 (30.7%)	930 (30.3%)
On dialysis	859 (30.0%)	536 (22.1%)	312 (14.0%)	59 (6.4%)
Significant pulmonary disease	1,637 (10.6%)	1,512 (16.0%)	1,191 (16.3%)	384 (12.5%)
Significant liver disease	535 (3.5%)	307 (3.3%)	129 (1.8%)	24 (0.8%)

All between-group comparisons were statistically significant (*P* < 0.001). Historical LVEF values were assessed only among patients with a prior diagnosis of heart failure (HF).

BMI = body mass index; LVEF = left ventricular ejection fraction.

### Admission diagnoses and indications

**Figure 1:**
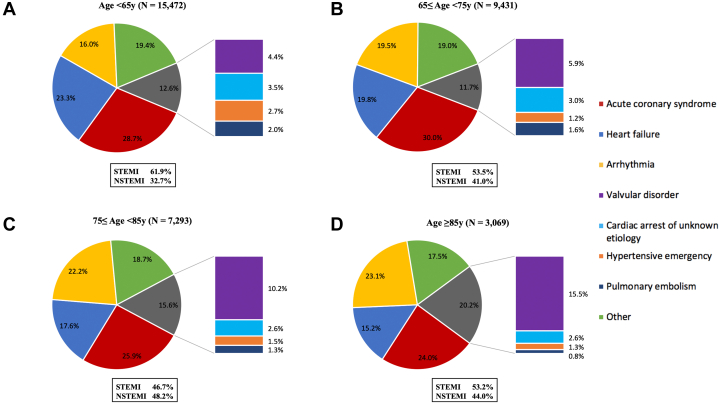
**Admission Diagnoses** Pie charts of the top 4 primary diagnoses of patients admitted to the CICU by age group (A) ≤65, (B) >65 to <75, (C) ≥75 to <85, and (D) ≥85 years old, expressed as a percentage of cases with available admission diagnoses. Acute coronary syndromes (ACS) were subdivided into STEMI and NSTEMI cases and depicted in an accompanying panel. NSTEMI = non–ST-segment elevation myocardial infarction; STEMI = ST-segment elevation myocardial infarction.

For all age groups, ACS was the most common primary diagnosis for CICU admission (Figure 1, Supplemental Table 2) with ST-segment elevation myocardial infarctions representing more than half of ACS cases among patients aged <75 years. There was a significantly higher burden of admission for valvular disease with increasing age. Valvular disease was the third most common reason for admission in the group aged ≥85 years (Figure 1). Post–transcatheter aortic valve implantation admissions were more common with advancing age, from 0.6% among patients aged <65 years to 12.5% among those aged ≥85 years (Supplemental Table 2) (*P* for trend <0.001). The proportion of admissions for arrhythmias also was higher with older age groups (Figure 1), with unstable conduction disorders comprising a progressively greater share among older patients, whereas ventricular tachyarrhythmias declined with advancing age (Supplemental Table 3). The proportion of admissions with CS decreased with increasing age, representing 22.5% and 12.6% of CICU indications among those <65 and ≥85 years, respectively (*P* < 0.001) (Table 2). Among patients with CS, the overall distribution of society for cardiovascular angiography and interventions stages was similar across age groups, with stage B most common and stage C the next most frequent. However, the oldest group (≥85 y) showed a relatively higher proportion of stages C and D (Supplemental Table 4).

**Table 2:** Indications for CICU Admission Among Patients in the Analysis Population (N = 35,265), Stratified by Age Group

ICU Indication	Age <65 y (n = 15,472)	Age ≤65 y to <75 y (n = 9,431)	Age ≤75 y to <85 y (n = 7,293)	Age ≥85 y (n = 3,069)	*P* Value
Respiratory insufficiency	3,709 (24.0%)	2,407 (25.5%)	1,885 (25.8%)	746 (24.3%)	0.005
Unstable arrhythmia	2,528 (16.3%)	1,952 (20.7%)	1,649 (22.6%)	742 (24.2%)	<0.001
Hypotension or shock					
Hypotension without shock	930 (6.0%)	634 (6.7%)	528 (7.2%)	202 (6.6%)	0.004
Cardiogenic shock	3,477 (22.5%)	1,931 (20.5%)	1,258 (17.2%)	388 (12.6%)	<0.001
Other shock	1,325 (8.6%)	963 (10.2%)	725 (9.9%)	293 (9.5%)	<0.001
Need for IV vasoactive therapy in absence of shock or hypotension	1,601 (10.3%)	809 (8.6%)	627 (8.6%)	282 (9.2%)	<0.001
Cardiac arrest	1,872 (12.1%)	1,063 (11.3%)	687 (9.4%)	216 (7.0%)	<0.001
Need for frequent labs or monitoring	2,978 (19.2%)	1,565 (16.6%)	1,061 (14.5%)	533 (17.4%)	<0.001
Postprocedural monitoring	1,601 (10.3%)	1,065 (11.3%)	1,058 (14.5%)	514 (16.7%)	<0.001
Need for ICU protocol medication or device	512 (3.3%)	303 (3.2%)	198 (2.7%)	51 (1.7%)	<0.001
Need for renal replacement therapy	658 (4.3%)	387 (4.1%)	245 (3.4%)	49 (1.6%)	<0.001
Neurological emergency	257 (1.7%)	142 (1.5%)	106 (1.5%)	34 (1.1%)	0.13
Other	209 (1.4%)	122 (1.3%)	57 (0.8%)	29 (0.9%)	0.001

Categories are mutually exclusive by hierarchical definition: 1) “Cardiogenic shock” excludes “other shock”; 2) “need for IV vasoactive therapy in absence of shock or hypotension” excludes “hypotension without shock,” “cardiogenic shock,” and “other shock”; 3) “need for ICU protocol medication or device” excludes all preceding categories; 4) “post-procedural monitoring” excludes all preceding categories plus “mechanical circulatory support”; and 5) “need for frequent labs or monitoring” excludes all preceding categories, including “postprocedural monitoring.”

IV = intravenous; ICU = intensive care unit; CICU = cardiac intensive care unit.

### Illness severity and advanced critical care

**Figure 2:**
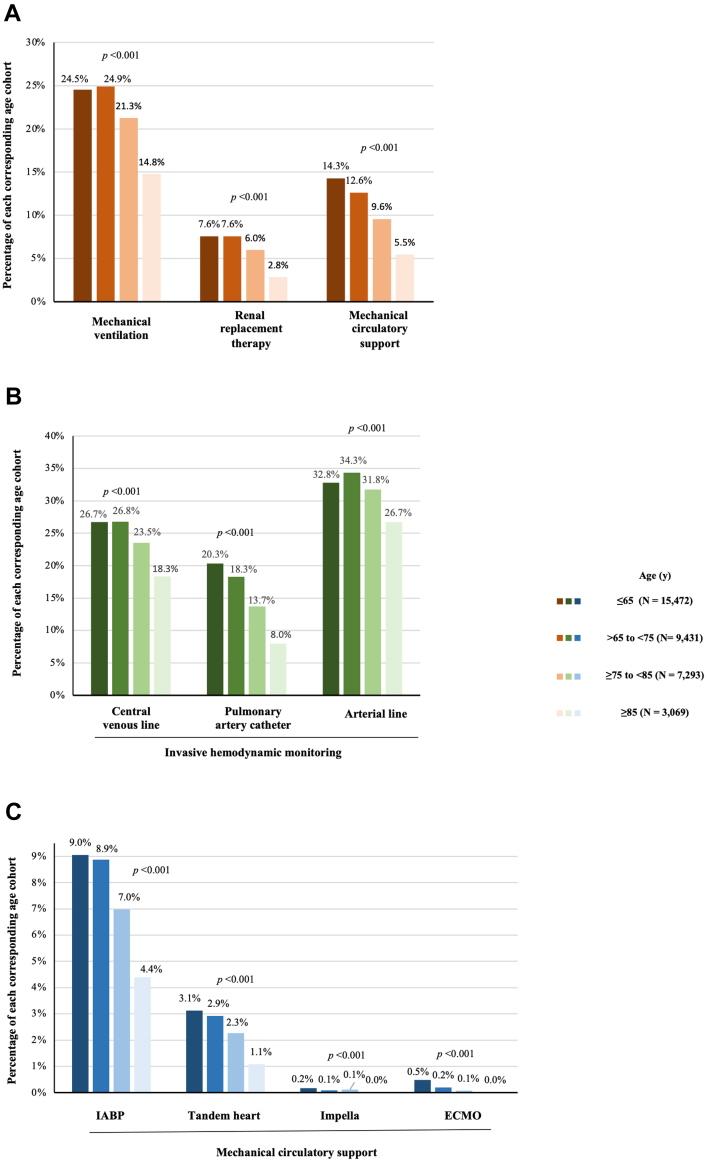
**Cardiac Intensive Care Unit Resource Utilization** (A) Resource utilization during the CICU admission in terms of mechanical ventilation, renal replacement therapy, invasive hemodynamic monitoring, and mechanical circulatory support use expressed as percentages of each corresponding age cohort in a bar chart configuration and compared among 4 age groups. (B) The frequency of use of different types of invasive hemodynamic monitoring and (C) mechanical circulatory support devices expressed as percentages of each corresponding age cohort in a bar chart configuration and compared among 4 age groups. All *P* values represent comparisons across all age groups. ECMO = extracorporeal membrane oxygenation; IABP = intra-aortic balloon pump.

Presentation SOFA scores and lactate levels were similar across age groups (Table 3). The oldest group (≥85 years) had the lowest median eGFR (45.2 mL/min/1.73 m^2^ [28.7-63.2]) (Table 3). The group aged ≥85 years used advanced CICU resources at lower rates compared with younger groups (Figure 2, Supplemental Table 5). The ≥85 years group had the lowest use of pulmonary artery catheters (8.0%), mechanical ventilation (14.8%), and mechanical circulatory support (MCS) (5.5%) (Figure 2). MCS platforms were least commonly used in the group ≥85 years. The only platform used in this age group was intra-aortic balloon pump. There were no patients in the ≥85 years group managed with extracorporeal membrane oxygenation (ECMO) or Impella (Figure 2C).

**Table 3:** Illness Severity, Worst Laboratory Values, and Duration of CICU Care Among Patients in the Analysis Population (N = 35,265), Stratified by Age Group

	Age <65 y (n = 15,472)	Age ≤65 y to <75 y (n = 9,431)	Age ≤75 y to <85 y (n = 7,293)	Age ≥85 y (n = 3,069)	*P* Value
Clinical features/illness severity					
SOFA score	3.0 (1.0-6.0)	4.0 (2.0-7.0)	4.0 (2.0-7.0)	4.0 (2.0-6.0)	<0.001
Worst laboratory values					
Lactate, median (IQR), mmol/L	2.2 (1.4-3.9)	2.2 (1.4-4.0)	2.1 (1.3-4.0)	2.0 (1.3-3.6)	0.001
Lactate >2 mmol/L	5,452 (52.5%)	3,382 (52.2%)	2,564 (50.8%)	1,020 (49.7%)	0.038
Arterial pH, median (IQR)	7.3 (7.2-7.4)	7.3 (7.2-7.4)	7.3 (7.2-7.4)	7.3 (7.3-7.4)	0.014
Arterial pH <7.25	810 (29.1%)	516 (30.4%)	352 (29.0%)	107 (24.9%)	0.17
eGFR, median (IQR), mL/min/1.73 m^2^	62.5 (34.0-86.1)	51.1 (27.2-73.8)	46.3 (26.3-68.3)	45.2 (28.7-63.2)	<0.001
eGFR <60 mL/min/1.73 m^2^	6,980 (47.8%)	5,365 (60.6%)	4,513 (66.3%)	2,017 (70.7%)	<0.001
ALT, median (IQR), U/L	36.0 (20.0-83.0)	32.0 (19.0-64.0)	28.5 (17.0-68.0)	25.0 (15.0-48.0)	<0.001
AST, median (IQR), U/L	47.0 (25.0-129.0)	42.0 (25.0-106.0)	41.5 (24.0-115.0)	35.0 (23.0-74.0)	<0.001
Total bilirubin, median (IQR), mg/dL	0.8 (0.5-1.4)	0.8 (0.5-1.3)	0.7 (0.5-1.2)	0.7 (0.5-1.0)	<0.001
Platelets, median (IQR), mg/dL	184.0 (129.0-239.0)	171.0 (123.0-222.0)	166.0 (123.0-217.0)	165.0 (126.0-214.0)	<0.001
INR, median (IQR)	1.2 (1.1-1.7)	1.3 (1.1-1.7)	1.3 (1.1-1.7)	1.2 (1.1-1.5)	0.002
Days of ICU care, median (IQR)	2.5 (1.2-5.6)	2.4 (1.2-5.2)	2.2 (1.1-4.5)	2.0 (1.1-3.8)	<0.001

ALT = alanine aminotransferase; AST = aspartate aminotransferase; eGFR = estimated glomerular filtration rate; INR = international normalized ratio; SOFA = sequential organ failure assessment; other abbreviations as in Table 2.

### Outcomes

Both CICU and hospital LOS were shorter with increasing age. The group aged >85 years had the shortest CICU LOS (2.0; IQR: 1.1-3.8 days) and overall hospital LOS (5.2; IQR: 2.7-9.7 days) (Supplemental Table 6). These trends remained largely consistent among patients with and without cardiac arrest, and the differences in in-hospital LOS across age groups persisted after multivariable adjustment for sex, illness severity (SOFA), and kidney function (Supplemental Table 7). In a sensitivity analysis excluding patients managed with comfort measures only, results for unadjusted LOS were largely unchanged (Supplemental Table 8).

CICU (13.0%) and in-hospital (17.8%) mortality rates were highest among the group aged 75 to 85 years (Table 4, Supplemental Table 9). After multivariable adjustment, the odds of in-hospital death increased progressively with age, appearing to plateau after age 75 years (Table 5). Compared with patients aged <65 years, adjusted ORs (95% CIs) for in-hospital mortality were 1.53 (1.40-1.67) for 65–<75 years, 1.83 (1.67-2.01) for 75–<85 years, and 1.95 (1.72-2.22) for ≥85 years. These findings remained consistent in a sensitivity analysis using multivariable Cox proportional hazards models to account for the timing of events and differences in LOS (Supplemental Table 10), another sensitivity analysis accounting for potential clustering by site using multivariable mixed-effects logistic regression (Supplemental Table 11), and in sensitivity analyses addressing the handling of missing lactate values using both complete case analysis and multiple imputation (Supplemental Table 12). The association between advancing age and in-hospital mortality also remained consistent in an expanded multivariable model that included additional adjustment for diabetes mellitus, significant pulmonary disease, and significant liver disease (Supplemental Table 13). A sensitivity analysis stratified by cardiac arrest status showed a similar trend among admissions without cardiac arrest, whereas in those presenting with cardiac arrest, the odds of in-hospital mortality rose more steeply with advancing age, reaching 3.09 (2.24-4.26) for patients ≥85 years (Table 5). An additional sensitivity analysis was performed stratified based on the presence of CS on admission. In the subgroup of patients with CS, CICU, and in-hospital mortality rates were higher across all age groups compared with the overall cohort (Supplemental Table 14). After multivariable adjustment, in-hospital mortality among patients with CS was higher with older age. Compared with patients aged <65 years, adjusted odds of in-hospital death were 1.51 (95% CI: 1.33-1.72) for those aged 65–<75 years, 2.21 (95% CI: 1.92-2.55) for 75–<85 years, and 2.24 (95% CI: 1.79-2.80) for ≥85 years. In analyses stratified by cardiac arrest status, similar age-associated higher rates of mortality were observed among patients without cardiac arrest, whereas among those presenting with both CS and cardiac arrest, the magnitude of the association with age was greater, with adjusted ORs of 1.69 (95% CI: 1.30-2.19), 3.29 (95% CI: 2.40-4.53), and 3.92 (95% CI: 2.32-6.63) for the 65–<75, 75–<85, and ≥85-year age groups, respectively (Supplemental Table 15).

**Table 4:** Unadjusted CICU and In-Hospital Mortality Among Patients in the Analysis Population (N = 35,265), Stratified by Age Group

	Age <65 y (n = 15,472)	Age ≤65 y to <75 y (n = 9,431)	Age ≤75 y to <85 y (n = 7,293)	Age ≥85 y (n = 3,069)
CICU N (% event rate)	1,259 (8.1%)	1,102 (11.7%)	950 (13.0%)	376 (12.3%)
In-hospital N (% event rate)				
All	1,785 (11.5%)	1,520 (16.1%)	1,295 (17.8%)	514 (16.7%)
Cardiac arrest	683 (34.4%)	493 (44.4%)	372 (51.5%)	133 (57.8%)
Non–cardiac arrest	1,102 (8.2%)	1,027 (12.3%)	923 (14.0%)	381 (13.4%)

All between-group comparisons were statistically significant (*P* < 0.001).

Abbreviation as in Table 2.

**Table 5:** Adjusted ORs for In-Hospital Mortality by Age Group Compared With Patients Aged <65 Years in the Analysis Population (N = 35,265), Stratified by Cardiac Arrest Status

	Comparison (Age Group vs <65 y)	Adjusted Difference in In-Hospital Mortality (95% CI)
All		
	65≤ Age <75 vs <65 y	1.53 (1.40-1.67)
	75≤ Age <85 vs <65 y	1.83 (1.67-2.01)
	Age ≥85 vs <65 y	1.95 (1.72-2.22)
Cardiac arrest		
	65≤ Age <75 vs <65 y	1.51 (1.27-1.80)
	75≤ Age <85 vs <65 y	2.11 (1.73-2.59)
	Age ≥85 vs <65 y	3.09 (2.24-4.26)
Non–cardiac arrest		
	65≤ Age <75 vs <65 y	1.55 (1.40-1.71)
	75≤ Age <85 vs <65 y	1.83 (1.64-2.04)
	Age ≥85 vs <65 y	1.90 (1.65-2.18)

Results are from multivariable logistic regression models adjusted for sex, illness severity (SOFA score), and kidney function (eGFR). All between-group comparisons were statistically significant (*P* < 0.001).

**Figure 3:**
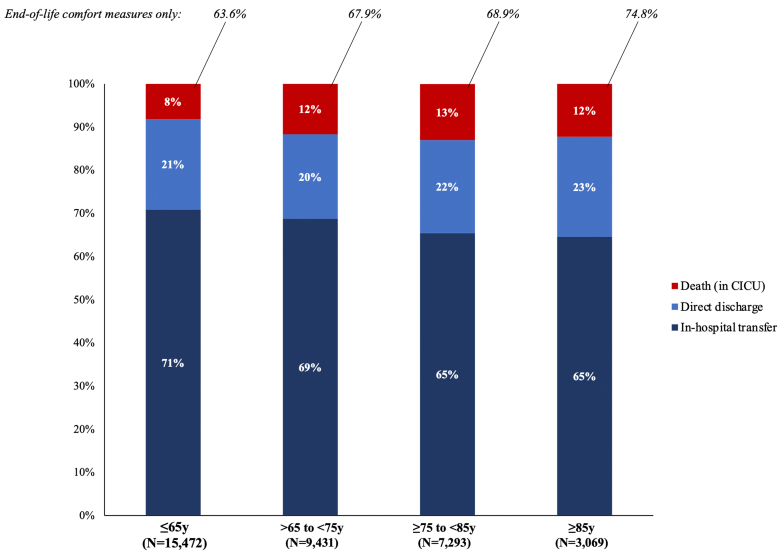
**Cardiac Intensive Care Unit Discharge Disposition** The CICU discharge disposition broken down into death (in CICU), in-hospital transfer (to a medicine or other floor), and direct discharge (from the CICU) in a stacked column configuration and compared among 4 age groups (*P* < 0.001 for all groups). Patients receiving end-of-life comfort measures only are noted as a percentage of the patients that died in the CICU instead of each age group in its entirety, with an observed significant (*P* < 0.001) increase in comfort measures use with increasing age. CICU = cardiac intensive care unit.

Discharge disposition from the CICU is presented in Figure 3. The oldest group (≥85 years) had the highest rate (23.2%, *P* < 0.001) of direct discharge from the CICU. Among patients who died, transition to comfort measures was more common among older age groups: 63.6%, 67.9%, 68.9%, and 74.8%, respectively (Figure 3). CICU and hospital discharge dispositions are further detailed in Supplemental Table 16, notable for progressively higher rates of hospital discharge to rehabilitation facilities with increasing age, peaking at 22.8% among patients aged ≥85 years. Code status at admission is presented in Supplemental Table 17 and was notable for a progressively higher prevalence of do-not-resuscitate/do-not-intubate status with advancing age (p for trend <0.001).

**Central Illustration:**
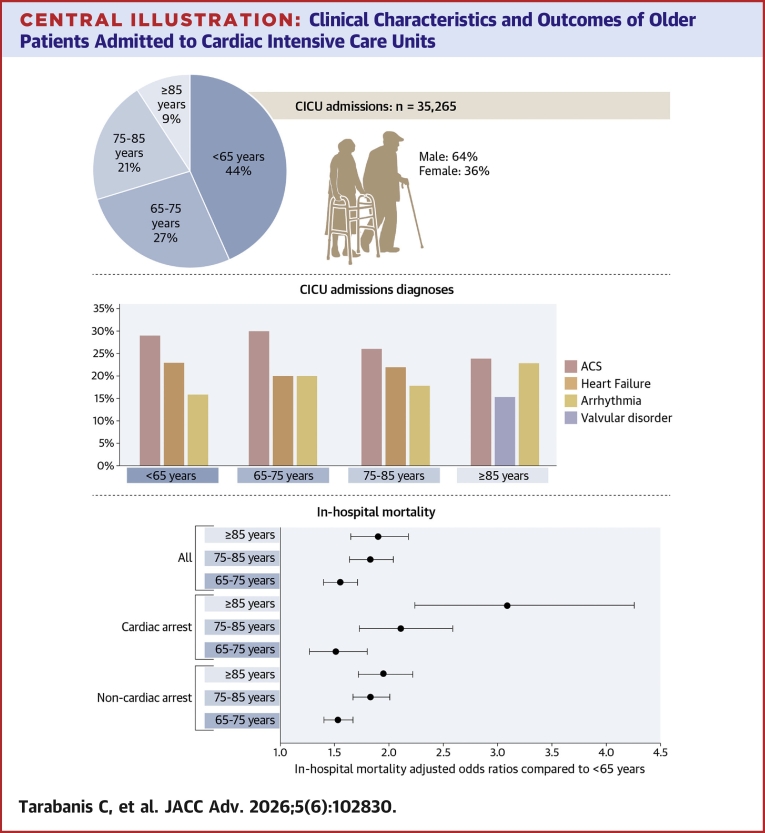
**Clinical Characteristics and Outcomes of Older Patients Admitted to Cardiac Intensive Care Units** The pie chart shows the age distribution of 35,265 consecutive medical CICU admissions (2017-2024) from the Critical Care Cardiology Trials Network (CCCTN) across 4 age groups (<65, 65–<75, 75–<85, and ≥85 years), along with the overall sex distribution (36% women and 64% men). The bar chart depicts the most common primary CICU admission diagnoses by age group, demonstrating increasing rates of valvular disease and arrhythmias with advancing age. The forest plot presents adjusted ORs (95% CIs) for in-hospital mortality compared with patients <65 years, overall and stratified by cardiac arrest and non–cardiac arrest admissions. Odds of death increased with advancing age and were highest among patients aged ≥85 years presenting with cardiac arrest, whereas among non–cardiac arrest admissions, adjusted mortality risk plateaued beyond 75 years. ACS = acute coronary syndrome.

## Discussion

This multicenter study provides a comprehensive, comparative analysis across age groupings of the clinical characteristics, advanced critical care therapies, and in-hospital outcomes of a large cohort of patients admitted to contemporary advanced CICUs with a wide spectrum of admission diagnoses and indications. Despite a greater burden of comorbid conditions, non–cardiac arrest patients aged 85 years or more who were triaged to cardiac critical care had rates of CICU survival (83%) that were qualitatively on par with patients aged 65 to 84 years. This finding contrasts with the preconceived notion that patients in that age group may have unacceptably higher risk of death in the CICU. Instead, it supports the concept that acceptable outcomes can be achieved for selected older patients undergoing cardiac critical care.

### Implications of age for cardiac critical care

Age is established as a major determinant of clinical outcomes in acute CV conditions and plays a role in decision making by clinicians. It is the single most common clinical parameter incorporated in risk stratification scores for patients with CS and other CV conditions.^20^^,^^21^ However, the role that age should play in triage to critical care remains to be established.^22^ The concept that age alone should not dictate intensive care triage decisions has been articulated by experts and professional societies.^23^^,^^24^ Such considerations might also apply to CICUs, but current evidence is limited, as prior studies have not systematically evaluated large cohorts of critically ill cardiac patients with advanced age; rather focused on specific conditions.^12^^,^^25^

In this context, our findings provide novel insight that enhances our understanding of an aging population with CVD, although addressing a gap in knowledge regarding practice patterns when caring for older patients in the CICU. We examined data from 19,793 CICU patients (56%) above the age of 65 years, nearly 1 in 11 (8.7%) of whom were ≥85 years old. Patients aged ≥85 years comprised a comparable proportion of medical ICU admissions in the BIDMC MIMIC-II database, where they accounted for 8.6% of all ICU admissions.^26^ We found that in-hospital mortality was similar (16% to 18%) across the cohort with ages 65 to >85 years. This observed in-hospital mortality rate among patients aged ≥85 years is similar to that in a prior single center retrospective study of 453 nonagenarians without a younger comparison group that reported a 15% in-hospital mortality.^7^

Accounting for illness severity and comorbidities, the adjusted odds of in-hospital death was higher in older patients relative to those <65 years but were comparable among age groups ≥65 years. The similar adjusted mortality across these age groups may reflect that biological age, rather than chronological age alone, drives outcomes. In other words, patients who survive into their late 80s and 90s, and who are deemed to reasonable for CICU admission, may represent a particularly robust subset despite advanced age. As well, clinicians are plausibly selecting patients >85 years with more favorable perceived prognosis as being appropriate for triage to critical care. This notion is reinforced by the observation that the overall use of advanced critical care therapies while in the CICU was lower for patients at the upper extreme of age. It is nevertheless important that it is possible in practice to identify such a cohort of patients with very advanced age with outcomes similar to those aged 65 to 75 years. Cardiac arrest, however, appears to be an exception to this pattern, as mortality was meaningfully higher with advancing age, suggesting that such an event may exceed the physiological reserve even of the most robust older patients.

Our findings highlight the challenges of appropriately evaluating critically ill older cardiac patients and underscore the need for a comprehensive assessment of each patient’s overall condition. Familiarity with geriatric syndromes, including multimorbidity, frailty, cognitive and physical decline, delirium, sensory impairment (hearing, vision, and pain), falls, and polypharmacy, should be taken into consideration when making CICU triage decisions.^27^^,^^28^ Clinicians should be aware that the incidence and prevalence of these syndromes can influence clinical presentation and correlate with prognosis.^29^^,^^30^ As well, shared decision-making for end-of-life care should include a comprehensive assessment of several factors rather than age alone, including the aforementioned geriatric syndromes, personal values and beliefs, local ethical, societal and cultural practices, institutional resources, as well as disease-specific parameters, such as cardiac arrest presentations.^3^^,^^31^

### CICU admission diagnoses and indications

Older patients presenting with ACS have been the focus of prior investigations and American Heart Association scientific statements.^12^, ^13^, ^14^, ^15^ In our study we similarly found that patients older than 75 years had comparatively higher rates of non-ST-segment elevation myocardial infarctions as their ACS presentation. This could be explained in part by the pathophysiological changes seen with advanced age, including increased aortic stiffness and impaired endothelial function, among others.^15^ Interestingly, prior studies focusing on ACS have shown mortality risk relationships that differ from the ones reported here. One single center retrospective study of 132 ACS patients admitted to the CICU reported higher in-hospital mortality rates among patients ≥80 years old (18.2% vs 3.2%, *P* = 0.0001).^12^ Similarly, data from community registries (e.g., National Cardiovascular Data Registry CathPCI Registry^32^) demonstrate increased mortality with advancing age, with the highest mortality rates observed among patients older than 80 to 85 years.^25^ This difference in mortality outcomes between prior studies and the present work indicates the importance of performing diagnosis-specific analyses. However, it also highlights a potential difference between older patients admitted to CICUs vs all-comers with advanced age receiving PCI, who might be monitored or treated in lower acuity units. Such potential explanations are supported in part by our data as we observed that as age increased, there was a higher proportion of patients most likely to be admitted to the CICU for postprocedural monitoring. In any case, overall ACS survival and specifically post-PCI in-hospital mortality rates have improved for all age groups over the past decade, but most notably for those older than 80 years.^25^^,^^33^^,^^34^

Regarding primary cardiac admission diagnoses other than ACS, our findings demonstrate that CICU admission rates for valvular disorders increased with increasing age, reaching 18% among patients aged ≥85 years. This finding tracks with the increasing age-adjusted prevalence of moderate to severe valvular heart disease in the general population (<2.0% vs 13.2% in <65 vs ≥75 years, respectively).^35^ These age-related trends in CICU admissions for valvular disease may carry particular relevance given recent data showing that, although valvular heart disease accounts for a minority of CS cases, it is associated with disproportionately high in-hospital mortality.^36^^,^^37^ Interestingly, the prevalence of heart failure with or without CS, as a reason for CICU admission, was lower with advancing age. This finding plausibly reflects selection in CICU admissions whereby priority is given to younger patients who might benefit from aggressive treatments (eg, invasive hemodynamics, MCS) or who might be considered candidates for bridging to advanced therapies, while older patients might have been triaged to non-CICU settings due to futility, advanced directives, or a transition to comfort measures, although such data were not captured in our study.

### Cardiogenic shock by age groups

In our study, the incidence of CS as a primary cardiac admission diagnosis as well as an indication for CICU-level of care decreased with advancing age. As little data exist on CICU resource utilization rates among older patients, the quantification of invasive hemodynamic monitoring and MCS use by age group is a novel aspect of this study. Unsurprisingly, we found that the use of invasive hemodynamic monitoring and MCS of all types, as well as of renal replacement therapy and mechanical ventilation decreased with advancing age. However, it is notable that no patients aged ≥85 years received Impella or ECMO, while still receiving intra-aortic balloon pumps for circulatory support. These findings suggest a judicious and comprehensive evaluation in clinical decision-making related to escalation of care, and limited potential bridging options from advanced MCS. Such practice patterns correlate with prior data both from an analysis of the National Inpatient Sample^38^ and a single center retrospective study,^39^ demonstrating higher mortality rates with advancing age among patients receiving venoarterial ECMO.^39^ Consistent with these patterns of selective use of advanced therapies, outcomes in patients with CS also varied by age. Although mortality increased with advancing age, particularly in those presenting with both CS and cardiac arrest, adjusted in-hospital mortality among noncardiac arrest CS patients leveled off beyond 75 years. This suggests that prognosis in older CS patients is influenced as much by the acuity of the presenting condition and treatment intensity as by chronological age itself.

### Study limitations

There are limitations to our study. First, by design, the registry only includes in-hospital outcomes. Secondly, the registry does not collect granular data regarding clinical decision-making surrounding triage and invasive intervention selection, representing an unmeasured variable that may have influenced the observed outcomes. In this respect, the findings support that clinicians are, on balance, successful in their selection of older patients for critical care therapies irrespective of age. Third, the lack of information relating to the frailty index and other risk markers previously validated in geriatric patients^7^ precludes the ability to examine frailty directly as a prognostic factor. Lastly, the observational nature of our data precludes causal inferences and the focus on primarily tertiary academic centers may limit generalizability to community-based ICUs.

## Conclusions

The present study expands our understanding of practice patterns in the care of older patients in the CICU. Patients aged ≥85 years selected for triage to the CICU generally had survival rates similar to patients aged 75 to 85 years, except in cases of cardiac arrest. This finding reinforces that age alone should not be relied on in triage decisions. Instead, clinicians should holistically examine older patients’ physiologic substrate recognizing that acceptable outcomes can be achieved among selected patients with very advanced age undergoing cardiac critical care.

Perspectives **COMPETENCY IN MEDICAL KNOWLEDGE:** Among critically ill cardiac patients ≥65 years old admitted to contemporary CICUs, in-hospital mortality increased with advancing age but plateaued beyond 75 years after adjustment for illness severity, with patients ≥85 years demonstrating survival comparable to those aged 75 to 85 years. These findings support the principle that chronological age alone should not serve as the sole consideration in CICU triage or treatment decisions.

**TRANSLATIONAL OUTLOOK:** Future research should delineate biological vs chronological contributors to outcomes in older CICU patients. Prospective studies are needed to evaluate how frailty assessment, structured triage pathways, and selective use of advanced therapies influence survival, functional recovery, and quality of life in older adults, particularly those presenting with CS or cardiac arrest.

## Funding support and author disclosures

Drs Morrow and Guo are members of the TIMI Study Group, which has received institutional research grant support through the Brigham and Women’s Hospital from Abbott, Abiomed, Amgen, Anthos Therapeutics, AstraZeneca, Bayer HealthCare Pharmaceuticals Inc, Daiichi-Sankyo, Eisai, Intarcia, Ionis Pharmaceuticals Inc, Janssen Research and Development LLC, Merck, Novartis, Pfizer, Quark Pharmaceuticals, Regeneron Pharmaceuticals Inc, Roche, Siemens Healthcare Diagnostics Inc, Softcell Medical Limited, and Zora Biosciences. Dr Morrow has received consulting fees from Abbott Laboratories, Merck & Co., Novartis, Regeneron, and Roche Diagnostics. Dr Fordyce reports consulting fees from Amgen, Boehringer Ingelheim, Novo Nordisk, Sanofi, HLS Therapeutics, Lilly, and Novartis and research grants from Amgen and Novo Nordisk. Dr Katz has received speaker honoraria from Abiomed and Zoll and nonfinancial research support from Abbott. Dr Miller has received an educational honorarium from Zoll. Dr Newby has received research grant support through Duke University from Roche Diagnostics, Medtronic, and BioKier and consulting fees from Medtronic and CSL. Dr Alviar has received consulting fees from Abott, Abiomed and 4TEEN4 and research support from Baxter Medical. All other authors have reported that they have no relationships relevant to the contents of this paper to disclose.
