# Supplementary material for: Clinical Characteristics and Outcomes of Older Patients Admitted to the Cardiac Intensive Care Unit
Source: JACC Adv. 2026 Jun 17;5(6):102830. doi: 10.1016/j.jacadv.2026.102830 (PMC13308241; doi:10.1016/j.jacadv.2026.102830)
Supplement: Supplemental_Material [file mmc1.docx]

**Supplementary Table 1.** Age-group composition of each CCCTN trial campaign in the analysis population (N=35,265), presented as frequencies, percentages, row percentages, and column percentages.

| **Campaigns** | **Frequency  Row percentage (%)** | | | | |
| --- | --- | --- | --- | --- | --- |
|  | **Age<65**  **N=15472** | **65<=Age<75 N=9431** | **75<=Age<85 N=7293** | **Age>=85 N=3069** | **Total** |
| **2017-18** | 2062  47.34 | 1125  25.83 | 811  18.62 | 358  8.22 | 4356 |
| **2018-19** | 1838  45.24 | 1105  27.20 | 763  18.78 | 357  8.79 | 4063 |
| **2019-20** | 2323  45.69 | 1356  26.67 | 1004  19.75 | 401  7.89 | 5084 |
| **2020-21** | 1947  43.03 | 1283  28.35 | 921  20.35 | 374  8.27 | 4525 |
| **2021-22** | 2375  42.33 | 1464  26.09 | 1239  22.08 | 533  9.50 | 5611 |
| **2022-23** | 2269  41.99 | 1453  26.89 15.41 | 1177  21.78 16.14 | 505  9.34 | 5404 |
| **2023-24** | 2658  42.72 | 1645  26.44 | 1378  22.15 | 541  8.69 | 6222 |
| **Total** | 15472 43.87 | 9431 26.74 | 7293 20.68 | 3069 8.71 | 35265 |

| **Characteristic** | **Age<65 n (%) N=15472** | **65<=Age<75 n (%) N=9431** | **75<=Age<85 n (%) N=7293** | **Age>=85 n (%) N=3069** | **P-value** |
| --- | --- | --- | --- | --- | --- |
| Acute coronary syndrome | 4441 (28.7%) | 2833 (30.0%) | 1887 (25.9%) | 737 (24.0%) | <0.001 |
| STEMI | 2747 (61.9%) | 1514 (53.5%) | 882 (46.7%) | 392 (53.2%) | <0.001 |
| NSTEMI | 1453 (32.7%) | 1161 (41.0%) | 909 (48.2%) | 324 (44.0%) | <0.001 |
| Heart failure | 3606 (23.3%) | 1871 (19.8%) | 1287 (17.6%) | 467 (15.2%) | <0.001 |
| Cardiogenic shock (etiology not o/w listed) | 804 (5.2%) | 381 (4.0%) | 238 (3.3%) | 61 (2.0%) | <0.001 |
| Heart failure | 2802 (18.1%) | 1490 (15.8%) | 1049 (14.4%) | 406 (13.2%) | <0.001 |
| Arrhythmia | 2480 (16.0%) | 1843 (19.5%) | 1616 (22.2%) | 710 (23.1%) | <0.001 |
| Ventricular arrhythmia | 1308 (8.5%) | 766 (8.1%) | 513 (7.0%) | 108 (3.5%) | <0.001 |
| Atrial arrhythmia | 697 (4.5%) | 458 (4.9%) | 347 (4.8%) | 129 (4.2%) | 0.365 |
| Unstable conduction disorder | 475 (3.1%) | 619 (6.6%) | 756 (10.4%) | 473 (15.4%) | <0.001 |
| Other | 4402 (28.5%) | 2436 (25.8%) | 2106 (28.9%) | 1014 (33.0%) | <0.001 |
| Valvular | 675 (4.4%) | 561 (5.9%) | 745 (10.2%) | 475 (15.5%) | <0.001 |
| Transcatheter aortic valve implantation | 98 (0.6%) | 270 (2.9%) | 586 (8.0%) | 385 (12.5%) | <0.001 |
| Cardiac arrest (unknown etiology) | 539 (3.5%) | 283 (3.0%) | 187 (2.6%) | 81 (2.6%) | <0.001 |
| Hypertensive Emergency | 416 (2.7%) | 114 (1.2%) | 107 (1.5%) | 41 (1.3%) | <0.001 |
| Pulmonary embolism | 307 (2.0%) | 147 (1.6%) | 96 (1.3%) | 25 (0.8%) | <0.001 |
| Aortic syndrome | 278 (1.8%) | 165 (1.7%) | 139 (1.9%) | 82 (2.7%) | 0.008 |
| Tamponade | 454 (2.9%) | 239 (2.5%) | 177 (2.4%) | 67 (2.2%) | 0.026 |
| General medical problem in patient with cardiac disease | 911 (5.9%) | 580 (6.1%) | 412 (5.6%) | 170 (5.5%) | 0.459 |
| Other | 822 (5.3%) | 347 (3.7%) | 243 (3.3%) | 73 (2.4%) | <0.001 |

**Supplementary Table 2.** Primary admission diagnoses among patients in the analysis population (N=35,265), stratified by age group. STEMI and NSTEMI counts were restricted to patients with acute coronary syndrome.

**Supplementary Table 3.** Subtypes of arrhythmia among patients with arrhythmia as the primary admission diagnosis in the analysis population (N=35,265), stratified by age group. Comparisons across age groups were statistically significant (p<0.001), except for atrial tachyarrhythmias (p=0.365).

|  | **Age<65 n (%) N=15472** | **65<=Age<75 n (%) N=9431** | **75<=Age<85 n (%) N=7293** | **Age>=85 n (%) N=3069** | **P-value** |
| --- | --- | --- | --- | --- | --- |
| Arrhythmia | 2480 (16.0%) | 1843 (19.5%) | 1616 (22.2%) | 710 (23.1%) | <0.001 |
| Ventricular tachycardia/fibrillation | 1308 (8.5%) | 766 (8.1%) | 513 (7.0%) | 108 (3.5%) | <0.001 |
| Atrial tachyarrhythmia | 697 (4.5%) | 458 (4.9%) | 347 (4.8%) | 129 (4.2%) | 0.365 |
| Unstable conduction disorder | 475 (3.1%) | 619 (6.6%) | 756 (10.4%) | 473 (15.4%) | <0.001 |

**Supplementary Table 4**. SCAI shock stage distribution among patients with cardiogenic shock and with sufficient data to determine stage (N=7,513), stratified by age group. Comparisons across age groups were statistically significant (p<0.05).

|  | **Age<65 n (%) N=3463** | **65<=Age<75 n (%) N=2133** | **75<=Age<85 n (%) N=1435** | **Age>=85 n (%) N=482** |
| --- | --- | --- | --- | --- |
| SCAI shock stage |  |  |  |  |
| A | 363 (10.5%) | 247 (11.6%) | 147 (10.2%) | 62 (12.9%) |
| B | 1984 (57.3%) | 1157 (54.2%) | 799 (55.7%) | 285 (59.1%) |
| C | 811 (23.4%) | 542 (25.4%) | 346 (24.1%) | 84 (17.4%) |
| D | 305 (8.8%) | 187 (8.8%) | 143 (10.0%) | 51 (10.6%) |

**Supplementary Table 5.** Use of advanced critical care resources among patients in the analysis population (N=35,265), stratified by age group. Comparisons across age groups were statistically significant (*p*<0.0001).

| **Resources** | **Age<65 n (%) N=15472** | **65<=Age<75 n (%) N=9431** | **75<=Age<85 n (%) N=7293** | **Age>=85 n (%) N=3069** |
| --- | --- | --- | --- | --- |
| Mechanical ventilation | 3794 (24.5%) | 2347 (24.9%) | 1550 (21.3%) | 453 (14.8%) |
| Renal replacement therapy | 1170 (7.6%) | 713 (7.6%) | 437 (6.0%) | 87 (2.8%) |
| Invasive monitoring | 6545 (42.3%) | 4059 (43.0%) | 2862 (39.2%) | 1020 (33.2%) |
| Central venous line | 4133 (26.7%) | 2528 (26.8%) | 1714 (23.5%) | 563 (18.3%) |
| Pulmonary artery catheter | 3147 (20.3%) | 1723 (18.3%) | 1000 (13.7%) | 245 (8.0%) |
| Arterial line | 5077 (32.8%) | 3239 (34.3%) | 2316 (31.8%) | 820 (26.7%) |
| Mechanical Circulatory Support | 2209 (14.3%) | 1191 (12.6%) | 698 (9.6%) | 168 (5.5%) |
| IABP | 1400 (9.0%) | 837 (8.9%) | 509 (7.0%) | 135 (4.4%) |
| Tandem heart | 483 (3.1%) | 275 (2.9%) | 165 (2.3%) | 33 (1.1%) |
| Impella | 27 (0.17%) | 8 (0.08%) | 8 (0.11%) | 0 (0%) |
| ECMO | 75 (0.48%) | 19 (0.20%) | 5 (0.07%) | 0 (0%) |
| Temporary surgical VAD | 0 (0%) | 1 (0.01%) | 0 (0%) | 0 (0%) |
| Unknown Device | 224 (1.45%) | 51 (0.54%) | 11 (0.15%) | 0 (0%) |

**Supplementary Table 6.** Unadjusted length of stay in the CICU and hospital among patients in the analysis population (N=35,265), stratified by age group. Comparisons across age groups were statistically significant (*p*<0.001).

| **Characteristic** | **Age<65 n (%) N=15472** | **65<=Age<75 n (%) N=9431** | **75<=Age<85 n (%) N=7293** | **Age>=85 n (%) N=3069** |
| --- | --- | --- | --- | --- |
| Unadjusted |  |  |  |  |
| CICU, Median (IQR), days | 2.5 (1.2-5.6) | 2.4 (1.2-5.2) | 2.2 (1.1-4.5) | 2.0 (1.1-3.8) |
| In-hospital, Median (IQR), days | 6.7 (2.9-14.6) | 6.6 (3.0-13.6) | 6.1 (2.9-11.9) | 5.2 (2.7-9.7) |
|  |  |  |  |  |
| In-hospital Mean (SD), days |  |  |  |  |
| All, Mean (SD), days | 12.7 (19.2) | 11.1 (14.8) | 9.7 (13.6) | 8.0 (12.0) |
| Cardiac arrest | 17.4 (19.9) | 16.3 (17.3) | 13.7 (11.9) | 11.0 (8.7) |
| Non-cardiac arrest | 12.2 (19.1) | 10.7 (14.5) | 9.4 (13.7) | 7.9 (12.1) |

**Supplementary Table 7.** Comparisons of in-hospital length of stay among patients in the analysis population (N=35,265) adjusted for sex, illness severity (SOFA score), and kidney function.

| **Characteristic** | **Comparison**  **(Age group vs <65y)** | **Adjusted difference in LOS (95% CI)** | ***p* value** |
| --- | --- | --- | --- |
| All |  |  |  |
|  | 65<=Age<75 vs. <65y | -1.89 (-2.33, -1.45) | <0.001 |
|  | 75<=Age<85 vs. <65y | -3.27 (-3.76, -2.78) | <0.001 |
|  | Age>=85 vs. <65y | -4.75 (-5.43, -4.08) | <0.001 |
| Cardiac arrest |  |  |  |
|  | 65<=Age<75 vs. <65y | -1.27 (-2.92, -0.38) | 0.131 |
|  | 75<=Age<85 vs. <65y | -3.49 (-5.53, -1.44) | 0.001 |
|  | Age>=85 vs. <65y | -5.71 (-9.26, -2.16) | 0.002 |
| Non-cardiac arrest |  |  |  |
|  | 65<=Age<75 vs. <65y | -1.94 (-2.39, -1.48) | <0.001 |
|  | 75<=Age<85 vs. <65y | -3.24 (-3.74, -2.74) | <0.001 |
|  | Age>=85 vs. <65y | -4.69 (-5.38, -4.01) | <0.001 |

**Supplementary Table 8.** Unadjusted length of stay in the CICU and hospital among patients in the analysis population excluding those managed with comfort measures only (N=32,154), stratified by age group. Comparisons across age groups were statistically significant (p<0.001).

| **Characteristic** | **Age<65 n (%) N=14456** | **65<=Age<75 n (%) N=8498** | **75<=Age<85 n (%) N=6484** | **Age>=85 n (%) N=2716** |
| --- | --- | --- | --- | --- |
| Unadjusted |  |  |  |  |
| CICU, Median (IQR), days | 2.4 (1.2-5.2) | 2.3 (1.1-4.9) | 2.1 (1.1-4.1) | 2.0 (1.1-3.7) |
| In-hospital, Median (IQR), days | 6.7 (2.9-14.6) | 6.6 (3.0-13.6) | 6.1 (2.9-11.9) | 5.2 (2.7-9.7) |
|  |  |  |  |  |
| In-hospital Mean (SD), days |  |  |  |  |
| All, Mean (SD), days | 12.7 (19.2) | 11.1 (14.8) | 9.7 (13.6) | 8.0 (12.0) |
| Cardiac arrest | 17.4 (19.9) | 16.3 (17.3) | 13.7 (11.9) | 11.0 (8.7) |
| Non-cardiac arrest | 12.2 (19.1) | 10.7 (14.5) | 9.4 (13.7) | 7.9 (12.1) |

**Supplementary Table 9.** Unadjusted odds ratios for CICU and in-hospital mortality among patients in the analysis population (N=35,265), stratified by age group. Comparisons across age groups were statistically significant (p<0.001).

| **Characteristic** | **Comparison** | **Odds ratio (95% CI)** |
| --- | --- | --- |
| CICU mortality |  |  |
|  | 65<=Age<75 vs. <65y | 1.49 (1.37 – 1.63) |
|  | 75<=Age<85 vs. <65y | 1.69 (1.55 – 1.85) |
|  | Age>=85 vs. <65y | 1.58 (1.39 – 1.78) |
| In-hospital mortality |  |  |
| All | 65<=Age<75 vs. <65y | 1.47 (1.37 – 1.59) |
| All | 75<=Age<85 vs. <65y | 1.65 (1.53 – 1.79) |
| All | Age>=85 vs. <65y | 1.54 (1.39 – 1.72) |
| Cardiac arrest | 65<=Age<75 vs. <65y | 1.52 (1.31 – 1.76) |
| Cardiac arrest | 75<=Age<85 vs. <65y | 2.02 (1.70 – 2.40) |
| Cardiac arrest | Age>=85 vs. <65y | 2.61 (1.98 – 3.44) |
| Non-cardiac arrest | 65<=Age<75 vs. <65y | 1.58 (1.45 – 1.73) |
| Non-cardiac arrest | 75<=Age<85 vs. <65y | 1.84 (1.67 – 2.02) |
| Non-cardiac arrest | Age>=85 vs. <65y | 1.74 (1.54 – 1.97) |

**Supplementary Table 10.** Multivariable Cox proportional hazards models for in-hospital mortality among patients in the analysis population (N=35,265), stratified by cardiac arrest status. Comparisons across age groups were statistically significant (p<0.001).

| **Characteristic** | **Comparison** | **Hazard ratio (95% CI)** |
| --- | --- | --- |
| All |  |  |
|  | 65<=Age<75 vs. <65y | 1.63 (1.51–1.77) |
|  | 75<=Age<85 vs. <65y | 2.11 (1.94–2.30) |
|  | Age>=85 vs. <65y | 2.65 (2.36–2.97) |
| Cardiac arrest |  |  |
|  | 65<=Age<75 vs. <65y | 1.42 (1.24–1.62) |
|  | 75<=Age<85 vs. <65y | 1.91 (1.65–2.21) |
|  | Age>=85 vs. <65y | 3.08 (2.50–3.79) |
|  |  |  |
| Non-cardiac arrest |  |  |
|  | 65<=Age<75 vs. <65y | 1.77 (1.61–1.95) |
|  | 75<=Age<85 vs. <65y | 2.29 (2.07–2.54) |
|  | Age>=85 vs. <65y | 2.76 (2.41–3.17) |

**Supplementary Table 11**. Multivariable mixed-effects logistic regression for in-hospital mortality accounting for clustering by site among patients in the analysis population (N=35,265), stratified by cardiac arrest status. Comparisons across age groups were statistically significant (p<0.001).

| **Characteristic** | **Comparison** | **Odds ratio (95% CI)** |
| --- | --- | --- |
| All |  |  |
|  | 65<=Age<75 vs. <65y | 1.63 (1.51–1.77) |
|  | 75<=Age<85 vs. <65y | 2.11 (1.94–2.30) |
|  | Age>=85 vs. <65y | 2.65 (2.36–2.97) |
| Cardiac arrest |  |  |
|  | 65<=Age<75 vs. <65y | 1.42 (1.24–1.62) |
|  | 75<=Age<85 vs. <65y | 1.91 (1.65–2.21) |
|  | Age>=85 vs. <65y | 3.08 (2.50–3.79) |
|  |  |  |
| Non-cardiac arrest |  |  |
|  | 65<=Age<75 vs. <65y | 1.77 (1.61–1.95) |
|  | 75<=Age<85 vs. <65y | 2.29 (2.07–2.54) |
|  | Age>=85 vs. <65y | 2.76 (2.41–3.17) |

**Supplementary Table 12.** Sensitivity analyses for in-hospital mortality comparing primary results with complete case and multiple imputation models for missing lactate data.

| **Age group comparison** | **Original Estimate** | **Complete Case** | **Imputation** |
| --- | --- | --- | --- |
| 65<=Age<75 vs. <65y | 1.53 (1.40-1.67) | 1.53(1.39-1.68) | 1.52 (1.40-1.66) |
| 75<=Age<85 vs. <65y | 1.83 (1.67-2.01) | 1.85 (1.67-2.04) | 1.84 (1.67-2.02) |
| Age>=85 vs. <65y | 1.95 (1.72-2.22) | 1.94 (1.69-2.23) | 1.95 (1.72-2.22) |

**Supplementary Table 13.** Expanded multivariable logistic regression model for in-hospital mortality adjusting for sex, illness severity, kidney function, lactate, and chronic comorbidities. Comparisons across age groups were statistically significant (p<0.001).

| **Characteristic** | **Comparison** | **Odds ratio (95% CI)** |
| --- | --- | --- |
| All |  |  |
|  | 65<=Age<75 vs. <65y | 1.49 (1.37–1.63) |
|  | 75<=Age<85 vs. <65y | 1.80 (1.64–1.98) |
|  | Age>=85 vs. <65y | 1.96 (1.72–2.22) |
| Cardiac arrest |  |  |
|  | 65<=Age<75 vs. <65y | 1.44 (1.20–1.72) |
|  | 75<=Age<85 vs. <65y | 2.02 (1.64–2.48) |
|  | Age>=85 vs. <65y | 3.10 (2.25–4.28) |
|  |  |  |
| Non-cardiac arrest |  |  |
|  | 65<=Age<75 vs. <65y | 1.52 (1.37–1.69) |
|  | 75<=Age<85 vs. <65y | 1.81 (1.63–2.01) |
|  | Age>=85 vs. <65y | 1.90 (1.65–2.19) |

**Supplementary Table 14.** Unadjusted CICU and in-hospital mortality among patients with cardiogenic shock in the analysis population (N=7,645), stratified by age group. Comparisons across age groups were statistically significant (p<0.001).

| **Characteristic** | **Age<65 n (%) N=3720** | **65<=Age<75 n (%) N=2111** | **75<=Age<85 n (%) N=1378** | **Age>=85 n (%) N=436** |
| --- | --- | --- | --- | --- |
| CICU N (% event rate) | 653 (17.6%) | 563 (26.7%) | 496 (36.0%) | 165 (37.8%) |
|  |  |  |  |  |
| Unadjusted |  |  |  |  |
| In-hospital N (% event rate) |  |  |  |  |
| All | 889 (23.9%) | 713 (33.8%) | 601 (43.6%) | 191 (43.8%) |
| Cardiac arrest | 331 (40.4%) | 245 (53.3%) | 195 (68.2%) | 59 (68.6%) |
| Non-cardiac arrest | 558 (19.2%) | 468 (28.3%) | 406 (37.2%) | 132 (37.7%) |

**Supplementary Table 15.** Comparisons of in-hospital mortality among patients with cardiogenic shock in the analysis population (N=7,645), stratified by age group. Comparisons across age groups were statistically significant (p<0.001).

| **Characteristic** | **Comparison**  **(Age group vs <65y)** | **Adjusted difference in mortality (95% CI)** |
| --- | --- | --- |
| All |  |  |
|  | 65<=Age<75 vs. <65y | 1.51 (1.33, 1.72) |
|  | 75<=Age<85 vs. <65y | 2.21 (1.92, 2.55) |
|  | Age>=85 vs. <65y | 2.24 (1.79, 2.80) |
| Cardiac arrest |  |  |
|  | 65<=Age<75 vs. <65y | 1.69 (1.30, 2.19) |
|  | 75<=Age<85 vs. <65y | 3.29 (2.40, 4.53) |
|  | Age>=85 vs. <65y | 3.92 (2.32, 6.63) |
| Non-cardiac arrest |  |  |
|  | 65<=Age<75 vs. <65y | 1.46 (1.26, 1.70) |
|  | 75<=Age<85 vs. <65y | 2.04 (1.73, 2.40) |
|  | Age>=85 vs. <65y | 2.00 (1.55, 2.57) |

**Supplementary Table 16.** CICU and hospital discharge disposition among patients in the analysis population (N=35,265), stratified by age group. Comparisons across age groups were statistically significant (p<0.001).

| **Characteristic** | **Age<65 n (%) N=15472** | **65<=Age<75 n (%) N=9431** | **75<=Age<85 n (%) N=7293** | **Age>=85 n (%) N=3069** |
| --- | --- | --- | --- | --- |
| CICU transfer/discharge disposition |  |  |  |  |
| Death (died in CICU) | 1259 (8.1%) | 1102 (11.7%) | 950 (13.0%) | 376 (12.3%) |
| CMO | 1016 (63.6%) | 933 (67.9%) | 809 (68.9%) | 353 (74.8%) |
| In-hospital transfer (transferred out of CICU) | 10969 (70.9%) | 6484 (68.8%) | 4773 (65.5%) | 1980 (64.5%) |
| Discharge from hospital (direct D/C from CICU) | 3244 (21.0%) | 1845 (19.6%) | 1569 (21.5%) | 713 (23.2%) |
|  |  |  |  |  |
| Hospital discharge disposition |  |  |  |  |
| Home | 11667 (75.4%) | 6336 (67.2%) | 4502 (61.7%) | 1820 (59.3%) |
| Rehab | 1808 (11.7%) | 1476 (15.7%) | 1408 (19.3%) | 701 (22.8%) |
| Death | 1785 (11.5%) | 1520 (16.1%) | 1295 (17.8%) | 514 (16.7%) |
| CMO | 1016 (63.6%) | 933 (67.9%) | 809 (68.9%) | 353 (74.8%) |
| Missing | 212 (1.4%) | 99 (1.0%) | 88 (1.2%) | 34 (1.1%) |

**Supplementary Table 17.** Code status at admission among patients in the analysis population with available code status data (N=26,846), stratified by age group. Patients with missing code status information (N=8,419) were excluded.

| **Code status** | **Age<65 n (%) N=11572** | **65<=Age<75 n (%) N=7201** | **75<=Age<85 n (%) N=5719** | **Age>=85 n (%) N=2354** |
| --- | --- | --- | --- | --- |
| Full code | 11412 (98.6%) | 6912 (95.6%) | 5154 (90.1%) | 1895 (80.5%) |
| Do not resuscitate (DNR) and/or  Do not intubate (DNI) | 156 (1.35%) | 283 (3.93%) | 555 (9.70%) | 454 (19.3%) |
| Comfort measures only (CMO) | 4 (0.03%) | 6 (0.08%) | 10 (0.17%) | 5 (0.21%) |
